# Expression and phosphorylation state analysis of intracellular protein kinases using Multi-PK antibody and Phos-tag SDS-PAGE

**DOI:** 10.1016/j.mex.2015.11.007

**Published:** 2015-11-19

**Authors:** Yasunori Sugiyama, Syouichi Katayama, Isamu Kameshita, Keiko Morisawa, Takuma Higuchi, Hiroshi Todaka, Eiji Kinoshita, Emiko Kinoshita-Kikuta, Tohru Koike, Taketoshi Taniguchi, Shuji Sakamoto

**Affiliations:** a Department of Life Sciences, Faculty of Agriculture, Kagawa University, Kagawa 761-0795, Japan; b Laboratory of Molecular Biology, Science Research Center, Kochi Medical School, Kochi 783-8505, Japan; c Department of Functional Molecular Science, Institute of Biomedical & Health Sciences, Hiroshima University, Hiroshima 734-8553, Japan

**Keywords:** Erk, extracellular-signal-regulated protein kinase, *λ*PPase, lambda protein phosphatase, PKA, cAMP-dependent protein kinase, Multi-PK antibody, Phos-tag, Protein kinase, Phosphorylation Signaling, Kinome

## Abstract

Protein kinase expression and activity play important roles in diverse cellular functions through regulation of phosphorylation signaling. The most commonly used tools for detecting the protein kinase are protein kinase-specific antibodies, and phosphorylation site-specific antibodies were used for detecting activated protein kinase. Using these antibodies, only one kinase was analyzed at a time, however, a method for analyzing the expression and activation of a panel of protein kinases in cells is not established. Therefore, we developed a combined method using Multi-PK antibody and Phos-tag SDS-PAGE for profiling the expression and phosphorylation state of intracellular protein kinases. Using the new method, changes in the expression and phosphorylation state of various protein kinases were detected in cells treated with anticancer agent which inhibit multiple tyrosine kinase activities. Therefore, the new method is a useful technique for analysis of intracellular protein kinases.

• Multi-PK antibody recognizes a wide variety of protein kinases in various species.
• Using Phos-tag SDS-PAGE, phosphorylated proteins are visualized as slower migration bands compared with corresponding non-phosphorylated proteins.
• This combined method can be used for detecting changes in the expression and phosphorylation state of various intracellular protein kinases.

## Graphical abstract

**Figure:**
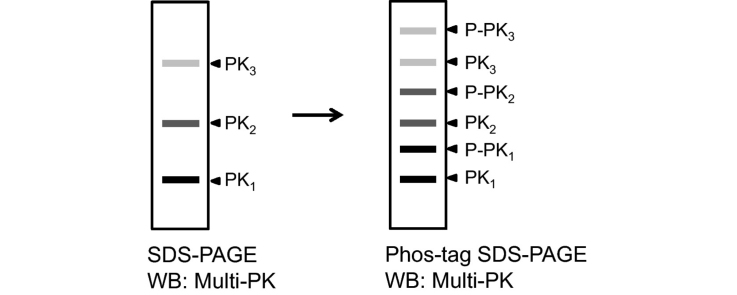

## Method details

Protein kinases are known to play pivotal roles in various cellular events through the regulation of diverse signaling pathways [1]. Therefore, it is important to know the expression and activity profiles of a panel of protein kinases under varying situations for elucidation of biological phenomena. To analyze the expression pattern of protein kinases in cells, we produced monoclonal antibodies, designated Multi-PK antibodies, to detect a wide variety of protein kinases [2], [3]. Meanwhile, phosphate-affinity SDS-PAGE (Phos-tag SDS-PAGE) was developed for mobility shift detection of phosphorylated proteins [4].

As many as 518 protein kinase genes have been identified in the human genome [5]. Although protein kinase expression patterns can be analyzed by Western blotting with Multi-PK antibodies, protein kinase activities cannot be detected using these antibodies. The activity of a large number of protein kinase is regulated by phosphorylation of a critical amino acid residue in an activation loop and/or regulatory domain of protein kinases through an upstream kinase or autophosphorylation [6]. Therefore, protein kinase activity is closely related to the phosphorylation state of protein kinases. In this article, we report a combined method for analysis of the expression and phosphorylation state of protein kinases by Western blotting analysis using Multi-PK antibodies after separation by Phos-tag SDS-PAGE.

### Materials

Recombinant mouse Src was expressed in *Escherichia coli* BL21 (DE3) and purified as described previously [3]. Recombinant human Abl1 and Lyn were purchased from Carna Biosciences. Anti-cAMP-dependent protein kinase (PKA) antibody, anti-phospho-PKA antibody, anti-extracellular-signal-regulated protein kinase (Erk)1/2 antibody and anti-phospho-Erk1/2 antibody were purchased from Cell Signaling Technology. Anti-Syk polyclonal antibody was obtained from Santa Cruz Biotechnology.

### Preparation of rat tissues extracts and protein determination

Rat tissues (cerebrum, cerebellum, medulla oblongata, heart, lung, stomach, small intestine, large intestine, spleen, liver, kidney, testis and muscle) were homogenized by Polytron homogenizer (Kinematica AG) in 10 volume of 5 mM Tris–HCl (pH 7.6) containing 0.5 mM EGTA, 1 mM EDTA and 1 mM phenylmethylsulfonyl fluoride. The homogenates were centrifuged at 20,000 × *g* for 30 min, and the supernatants thus obtained were used as crude extracts. Proteins were determined by the method of Bensadoun and Weinstein [7] using BSA as a standard. The samples were added to an equal volume of 2× SDS-PAGE sample buffer and boiled for 10 min, and subjected to SDS-PAGE.

### Cell culture and treatment of agents

INS-1 and HL-60 cells were cultured as described previously [3], [8]. INS-1 cells were cultured in serum free RPMI1640 for 6 h to starve the cells, and induced to change their intracellular phosphorylation signaling in the presence of 10 μM Forskolin, activator of Erk1/2 thorough PKA activation, for 10 min or 1 μM okadaic acid, an inhibitor of protein serine/threonine phosphatases PP1 and PP2A, for 1 h in 10-cm dishes. HL-60 cells were incubated with 10 μM anticancer agents (multiple tyrosine kinase inhibitors: axitinib, cabozantinib, pazopanib, vandetanib) for 3 days in 10-cm dishes. After incubation, the cells were mixed with 150 or 500 μl of SDS-PAGE sample buffer. The cell lysates were dephosphorylated by lambda protein phosphatase (*λ*PPase) as described previously [9], and precipitated by adding an equal volume of 20% trichloroacetic acid. The pellets were washed with ice-cold acetone, and dissolved in 2× SDS-PAGE sample buffer and boiled for 10 min, and analyzed by SDS-PAGE or Phos-tag SDS-PAGE followed by Western blotting.

### SDS-PAGE, Phos-tag SDS-PAGE and Western blotting

The 20 or 50 μg cell lysates were subjected to SDS-PAGE or Phos-tag SDS-PAGE. The SDS-polyacrylamide gels consisted of an 8% or 10% acrylamide separation gel and a 3% stacking gel. The Phos-tag gels consisted of a separating gel copolymerized with Phos-tag (6% acrylamide, 20 μM acrylamide-pendant Phos-tag, 40 μM MnCl_2_). After electrophoresis, Phos-tag gels were soaked in a transfer buffer (25 mM Tris, 192 mM glycine, and 20% methanol) containing 1 mM EDTA for 20 min with gentle agitation for elimination of the manganese ions from the gel. Next, the gels were soaked in a transfer buffer without EDTA for 10 min with gentle agitation. The resolved proteins were electrophoretically transferred to nitrocellulose membranes (GE Healthcare Biosciences, Protran BA85). The membranes were incubated with Multi-PK antibody (YK34), anti-phospho-PKA antibody, anti-Syk antibody diluted 1:200 or an anti-PKA antibody, anti-Erk1/2 antibody, anti-phospho-Erk1/2 antibody diluted 1:1000 for 2 h at room temperature. The membranes were then incubated with horseradish peroxidase conjugated anti-mouse IgG (Sigma) diluted 1:2000 or horseradish peroxidase conjugated anti-rabbit IgG (Cappel) diluted 1:5000 for 1 h, and the immunoreactive bands were detected using the chemiluminescent substrate, Western Lightning Plus-ECL (PerkinElmer).

## Method validation

**Fig. 1:**
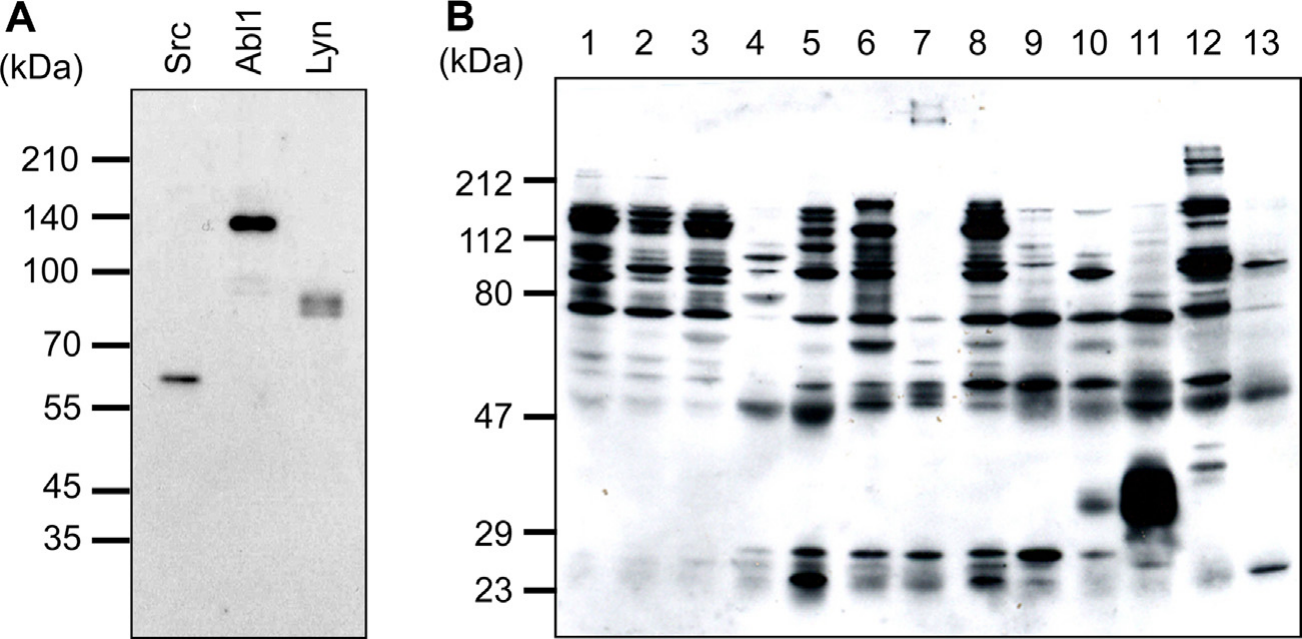
Reactivity of YK34 antibody by Western blotting. Approximately 50 ng of recombinant tyrosine kinases, Src, Abl1 and Lyn (A), and 20 μg of rat tissue extracts, cerebrum (lane 1), cerebellum (lane 2), medulla oblongata (lane 3), heart (lane 4), lung (lane 5), stomach (lane 6), small intestine (lane 7), large intestine (lane 8), spleen (lane 9), liver (lane 10), kidney (lane 11), testis (lane 12) and muscle (lane 13) (B) were electrophoresed on SDS-PAGE. The protein bands were detected immunologically by YK34 antibody.

In our earlier study, we produced unique monoclonal antibody YK34 directed to a highly conserved region (subdomain VIB) of tyrosine kinases. This antibody recognized various subdomain sequences in many of tyrosine kinases [3]. To evaluate usefulness of YK34 antibody, we examined cross-reactivity of this antibody using several recombinant tyrosine kinases. Three tyrosine kinases, Src, Abl1 and Lyn, were detected by Western blotting with YK34 antibody (Fig. 1A). In addition, when rat various tissue extracts were analyzed using YK34 antibody, many immunoreactive bands were observed (Fig. 1B). In our previous study, we showed that this antibody could recognize more than 75% of tyrosine kinases by analysis using Src mutants possessing different subdomain VIB sequences [3]. These results, taken together, suggested that YK34 antibody will be a powerful tool for detecting a variety of tyrosine kinases.

**Fig. 2:**
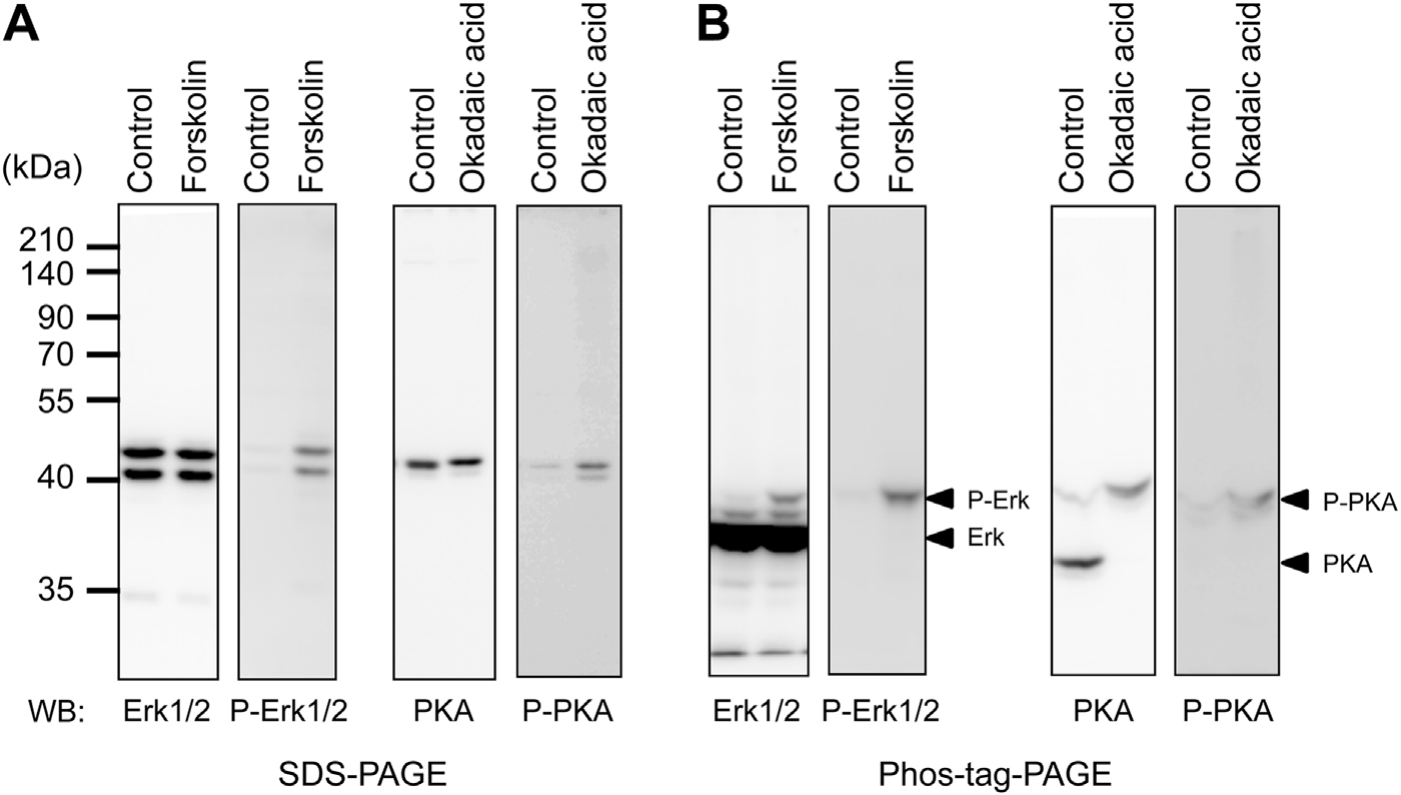
Detection of up-shifted band of phosphorylated protein kinases by Phos-tag SDS-PAGE. INS-1 cells were stimulated with 10 μM Forskolin or 1 μM okadaic acid. The cell extracts (20 or 50 μg) were separated by SDS-PAGE (A) or Phos-tag SDS-PAGE (B), and immunoreactive bands were detected by Western blotting using anti-Erk1/2 antibody, anti-phospho-Erk1/2 antibody, anti-PKA antibody and anti-phospho-PKA antibody.

Next, we examined whether phosphorylation-dependent activated protein kinases were detected as up-shifted bands compared with corresponding non-phosphorylated protein kinases using Phos-tag SDS-PAGE. When INS-1 cells were stimulated by Forskolin and okadaic acid, the phosphorylated bands of Erk1/2 and PKA were detected by Western blotting using phosphorylation of activation site-specific antibodies after separation by SDS-PAGE (Fig. 2A). On the other hand, when using Phos-tag SDS-PAGE, phosphorylated Erk1/2 and PKA were detected as up-shifted bands shown in stimulated cells (Fig. 2B). These results suggested that phosphorylated protein kinases were detected as slower migration bands compared with non-phosphorylated kinases.

**Fig. 3:**
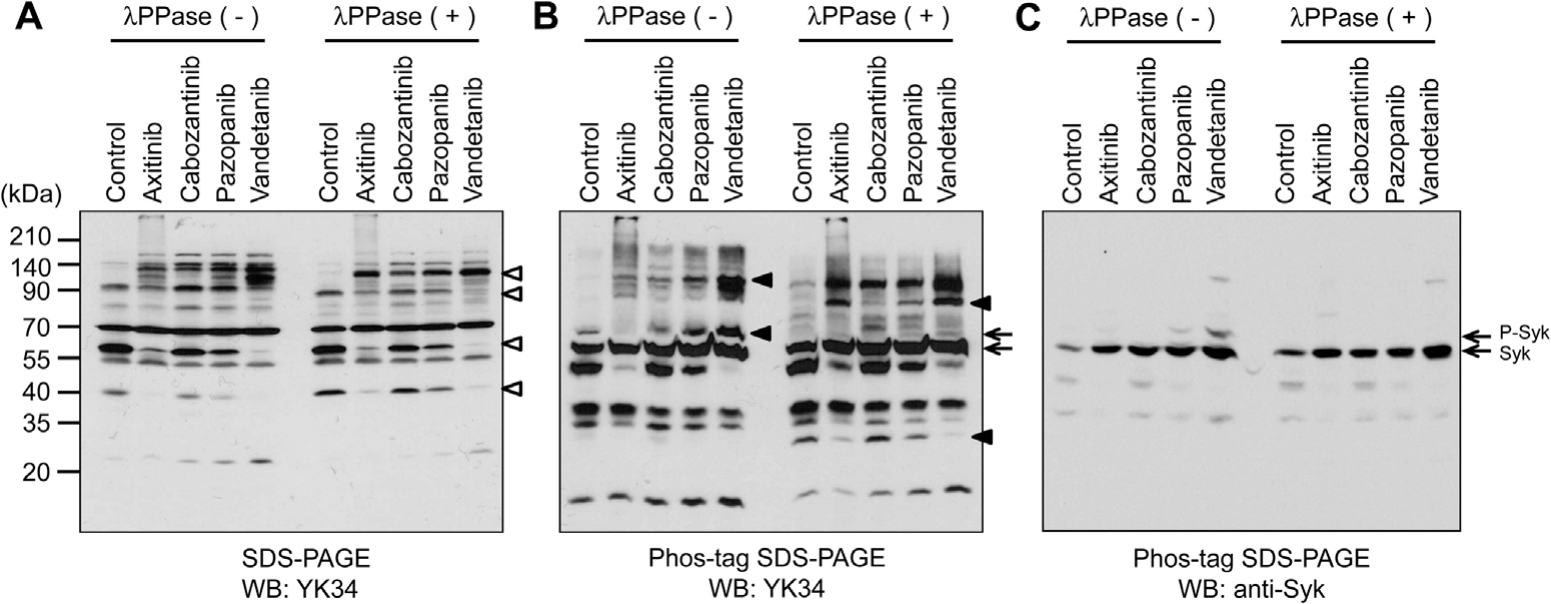
Expression and phosphorylation profiles of tyrosine kinases in HL-60 cells treated with anticancer agents. HL-60 cells were treated with 10 μM axitinib, cabozantinib, pazopanib or vandetanib for 3 days. The cell extracts (20 μg) were separated by SDS-PAGE (A) or Phos-tag SDS-PAGE (B, C). Immunoreactive bands were detected by Western blotting using YK34 antibody (A, B) or anti-Syk antibody (C). Open arrowheads indicate bands showing altered expression. Closed arrowheads indicate bands showing altered phosphorylation. Arrows indicate the migration positions of phosphorylated and non-phosphorylated Syk.

In recent studies, protein kinase inhibitors such as anticancer agents were shown to induce dynamic changes in protein kinase expression and activity in mammalian cells [10], [11]. Here, we attempted to detect the changes in protein kinase expression and phosphorylation state in human promyelocytic leukemia HL-60 cells by treatment with the anticancer agents, axitinib, cabozantinib, pazopanib, and vandetanib, which are multiple tyrosine kinase inhibitors. In Western blotting analysis using YK34 after separation by SDS-PAGE, various immunoreactive bands were detected and the protein levels of four bands were significantly changed by anticancer agents (Fig. 3A, open arrowheads). Moreover, when using Phos-tag SDS-PAGE, the immunoreactive bands patterns from anticancer agents treated HL-60 cells were markedly changed and these band patterns were altered by *λ*PPase treatment (Fig. 3B, closed arrowheads). The cross-reactive bands shown by arrows in Fig. 3B might be Syk, because similar changes were observed when Western blotting analysis was carried out using anti-Syk antibody (Fig. 3C, arrows). Therefore, Syk appeared be one of the potential targets of vandetanib in HL-60 cells. These results suggested that expression and phosphorylation state of tyrosine kinases in HL-60 cells were changed by treatment of anticancer agents.

## Additional information

In the present study, our findings show that the method of combination of Multi-PK antibody and Phos-tag SDS-PAGE will be a powerful technique for analysis of the expression and phosphorylation state of protein kinases in human cells. This method is especially useful for the first screening to explore the responsive kinases involved in various biological processes, though the identities of protein kinases cannot be clarified by this procedure. Consequently, when protein kinases of interest were detected by the first screening, these proteins could be identified by means of LC–MS/MS analysis or other methods that we have developed previously [8], [12], [13], [14].

In our previous studies, we established three hybridoma cell lines (M1C, M8C, YK34) which produce Multi-PK antibody [2], [3]. M1C and M8C antibodies recognized serine/threonine kinases and YK34 antibody directed to tyrosine kinases. These antibodies recognized various protein kinases in human [3], mouse [2], *Xenopus laevis* [15], zebrafish *Danio rerio* [16], plant *Lotus japonicus* [17], mushroom *Coprinopsis cinerea* [18]. Furthermore, using these antibodies, it was shown that cyclin-dependent kinase-like 5 bound and phosphorylated DNA methyltransferase 1 [14], Calcium/calmodulin-dependent protein kinase IV was involved in the pathophysiology of glucotoxicity [8], and the expression level of focal adhesion kinase was increased in oxazolone-induced epithelial cells of colitis model [19]. Therefore, we believe that this technique can be used for the elucidating of biological phenomena and disease pathogenesis through analysis of intracellular phosphorylation signaling in a wide variety biological species.
